# A new conceptual framework for maternal morbidity

**DOI:** 10.1002/ijgo.12463

**Published:** 2018-05-23

**Authors:** Veronique Filippi, Doris Chou, Maria Barreix, Lale Say, the WHO Maternal Morbidity Working Group (MMWG), Kelli Barbour, Jose G. Cecatti, Maria L. Costa, Sara Cottler, Olubukola Fawole, Tabassum Firoz, Luis Gadama, Atf Ghérissi, Gathari N. Gichuhi, Gill Gyte, Michelle Hindin, Anoma Jayathilaka, Amanda Kalamar, Marge Koblinsky, Yacouba Kone, Nenad Kostanjsek, Isabelle Lange, Laura A. Magee, Arvind Mathur, Affette McCaw‐Binns, Stephen Munjanja, Max Petzold, Elizabeth Sullivan, Frank Taulo, Özge Tunçalp, Rachel Vanderkruik, Peter von Dadelszen, Mark Morgan

**Affiliations:** ^1^ Department of Infectious Disease Epidemiology London School of Hygiene and Tropical Medicine London UK; ^2^ UNDP–UNFPA–UNICEF–WHO–World Bank Special Programme of Research Development and Research Training in Human Reproduction (HRP) Department of Reproductive Health and Research WHO Geneva Switzerland

**Keywords:** Conceptual framework, Health‐related functioning, Maternal morbidity, Pregnancy complications, Quality of life

## Abstract

**Background:**

Globally, there is greater awareness of the plight of women who have complications associated with pregnancy or childbirth and who may continue to experience long‐term problems. In addition, the health of women and their ability to perform economic and social functions are central to the Sustainable Development Goals.

**Methods:**

In 2012, WHO began an initiative to standardize the definition, conceptualization, and assessment of maternal morbidity. The culmination of this work was a conceptual framework: the Maternal Morbidity Measurement (MMM) Framework.

**Results:**

The framework underscores the broad ramifications of maternal morbidity and highlights what types of measurement are needed to capture what matters to women, service providers, and policy makers. Using examples from the literature, we explain the framework's principles and its most important elements.

**Conclusions:**

We express the need for comprehensive research and detailed longitudinal studies of women from early pregnancy to the extended postpartum period to understand how health and symptoms and signs of ill health change. With respect to interventions, there may be gaps in healthcare provision for women with chronic conditions and who are about to conceive. Women also require continuity of care at the primary care level beyond the customary 6 weeks postpartum.

## Abstract 2

A new Maternal Morbidity Measurement framework is presented, highlighting the consequences of maternal morbidity and the measurement required to capture the needs of women, providers, and policy makers.

## 1 Introduction

Over the last 15 years, maternal mortality has declined in most parts of the world, although not as much as anticipated when the Millennium Development Goals (MDGs) were agreed to in 2000. The maternal morbidity burden also remains substantial, especially in comparison with mortality, although estimates vary. Graham et al.^1^ calculated, for example, 27 million morbidity episodes for the five most common direct obstetric complications alone (eclampsia, pre‐eclampsia, postpartum hemorrhage, puerperal infection, and abortion complications) in 2015.

In view of these large numbers, there is a greater awareness at the global level of the plight of women who have complications associated with pregnancy or childbirth and who may continue to have problems in the long term. Fortunately, the health of women and the ability of women to perform economic and social functions are a central concept in the Sustainable Development Goals (SDGs)^2^ and there have been calls for “rethinking maternal health” using a life cycle or life‐course approach.^3^ In particular, the “Survive, Thrive and Transform” agenda of the “Global Strategy for Women's, Children's and Adolescents’ Health (2016–2030)”^4^ moves away from a single focus on maternal and child mortality reduction, by adding an emphasis on ensuring good health so that women, adolescents, and children can play their full role in future development.

**Figure 1:**
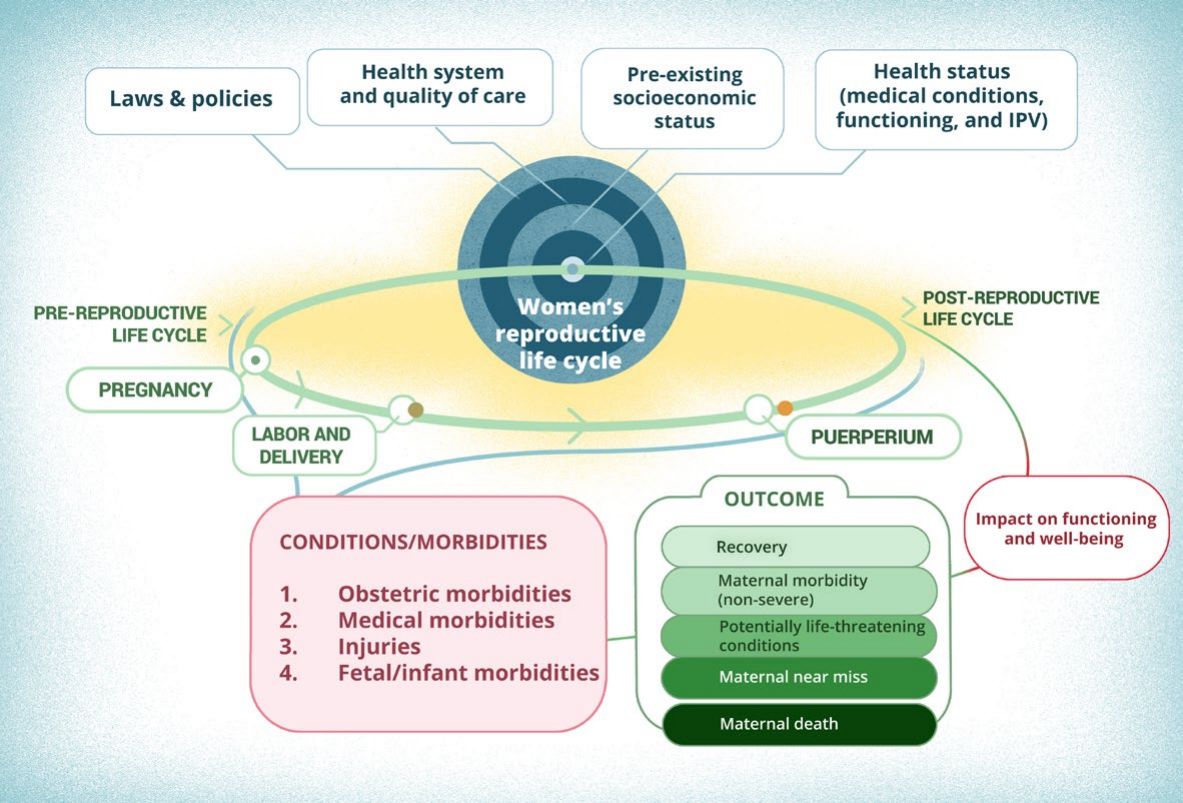
Maternal morbidity measurement (MMM) framework.

In 2012, WHO began a program of work on the definition, conceptualization, and assessment of maternal morbidity. The work aimed to document the various definitions of maternal morbidity and to develop a common approach for better understanding its magnitude. A Maternal Morbidity Working Group (MMWG) was established to work on this challenging agenda^5^ and focused on understanding the entire experience of morbidity, including non‐life‐threatening conditions as well as more severe ones. The MMWG agreed on the following definition of maternal morbidity: “any health condition attributed to and/or complicating pregnancy and childbirth that has a negative impact on the woman's wellbeing and/or functioning”.^6^ This work led to a conceptual framework, entitled the Maternal Morbidity Measurement (MMM) Framework, externally reviewed, and displayed in Figure 1.

## 2 Aims of the MMM Framework

This conceptual framework underscores the broad ramifications of maternal morbidity and highlights the types of measurement that should take place to capture everything that matters to women, service providers, and policy makers. The framework is also expected to have important implications for healthcare interventions and programs, which are explored in the article by Firoz et al.^7^ in this Supplement. A better understanding of maternal morbidity should, in due course, lead to a lesser burden as better policies are put in place and tailored services are provided. The novelty of the conceptual framework, and the associated definition of maternal morbidity, is that for maternal health it accounts for and applies the principles of the WHO International Classification of Functioning, Disability and Health (ICF).

## 3 Why A New Conceptual Framework?

Despite the large numbers of estimated complications mentioned earlier, there is little comprehensive research on maternal morbidity. The range of conditions is so large^6^ that studies often focus on the most life‐threatening or debilitating causes of obstetric morbidity and/or on a single condition.^8^ The lack of comprehensiveness is not just in terms of conditions, but also in relation to the duration of observation. Cross‐sectional surveys of self‐reported symptoms of ill health during pregnancy or the postpartum period exist,^9^ but cohort studies that integrate both diagnoses and self‐reports of ill health are rare.^10^ In 1999, Fortney and Smith^11^ noted “the literature is replete with hospital‐based studies, case studies and anecdotes describing acute and chronic morbidities with pregnancy and delivery. What does remain relatively unknown is the prevalence of morbidity—specific or general—in the population as a whole.” These remarks remain largely true today; little has changed in terms of morbidity evidence despite the increase in data on maternal health.^8^, ^12^

The dearth of comprehensive research and data is a result of both difficulties with measurement approaches and a lack of systematic understanding of the many facets of maternal morbidity. Concerning measurement, in the early 1990s, researchers were initially hopeful that they could capture robust population‐based information on direct obstetric morbidity in retrospective interview surveys. These attempts failed in low‐income countries as the lack of specificity of questionnaires led to an overestimation of maternal morbidity,^13^ although some successes were registered in higher‐resourced settings.^14^ Most published work on maternal morbidity has since focused on near‐miss morbidity identified in health facilities,^15^, ^16^ chronic conditions of importance to other medical specialties (i.e. psychiatry), and long‐term debilitating conditions such as vesicovaginal fistulae, associated with the ongoing Campaign to End Fistula. The lack of rich and comprehensive information is found not only in quantitative studies of maternal morbidity but also in qualitative ones.

The recently published literature suggests that research efforts are expanding their focus. There is a greater awareness of the contribution of chronic conditions and indirect causes of mortality to the burden of ill health experienced by women, most notably the contribution of diabetes.^17^ Researchers have started documenting economic and social consequences of maternal morbidity, and their relationship with productivity, economic growth, and development.^18^ A recent paper, fittingly entitled “Minor ailments in pregnancy are not a minor concern for pregnant women” has shown that nine out of 10 women disclosed at least one episode of ill health during pregnancy in Sri Lanka and that 60% of women could not do their day‐to‐day and work‐related activities because of ill health.^19^ There is also a greater awareness of the importance of assessing functioning and well‐being in relation to health conditions, including maternal complications.^20^, ^21^ Health‐related functioning refers to all bodily functions (physical and cognitive), activities, and participation. It is the positive correlate of disability—a term more frequently used in older maternal morbidity literature. Well‐being, on the other hand, relates to patients’ satisfaction with their health status and is measured with quality‐of‐life instruments.

## 4 Previous conceptual frameworks

As mentioned earlier, the aim of the MMM Framework is to highlight the implications of maternal morbidity by describing its many facets in detail and promoting better measurement. Prevailing conceptual frameworks in maternal health have different objectives. They concentrate mostly on risk factors for maternal mortality and interventions to reduce it, and not particularly on the experience and consequences of maternal deaths for families and communities. The most well‐known frameworks describe the distal and intermediate determinants of maternal mortality^22^ and the role of emergency obstetric care using the popular “three delays model”.^23^ Other frameworks address the analysis of the quality of care and health systems issues in relation to audits of maternal deaths and severe morbidity cases.^24^ While authors of these conceptual frameworks indicate that they can be readily applied to life‐threatening conditions, they do not unpack the concept of morbidity, especially non‐severe morbidity. Existing discussions or conceptual frameworks specific to maternal morbidity have emphasized the “base of the iceberg”^25^; the determinants of fistulae formation, including clinical determinants^26^; the various stages in the severity of maternal morbidity (WHO); or the consequences, disabilities, or sequelae attached to life‐threatening morbidity for mothers, babies, and households.^27^, ^28^

## 5 Principles of the MMM Framework

The MMM Framework reflects six key principles:

1. The importance of using a woman‐centered approach. In other words, women's perspectives on what is important to them regarding their health. As alluded to earlier, health problems that stop women from performing their normal activities may have substantial direct or indirect impact on their lives and on other members of their households. A woman‐centered approach is also why the framework includes adverse fetal and infant outcomes, as these can lead to an adverse maternal outcome. There is ample evidence, for example, that stillbirths are linked to psychological distress,^29^ and women who experience stillbirth may take longer than other women to recover from complications.
2. Maternal morbidity risks are cyclical since women can become pregnant more than once. In addition, sequelae of a maternal condition can occur in the next pregnancy. Women who deliver by cesarean, for example, are at increased risk of placenta previa in subsequent pregnancies.^30^
3. The effects of maternal morbidity can last a long time, beyond the customary 6 weeks postpartum, and there may be consequences later in life, during the postreproductive or postmenopausal periods. For instance, women who have hypertension during pregnancy are more likely to suffer cardiovascular diseases at older ages.
4. Maternal health is a social and economic phenomenon, not just a clinical and biological issue.
5. Context and environment influence the lived experience of morbidity. Living in a supportive environment can lead to better outcomes.
6. Finally, the framework includes meaningful groupings of maternal morbidity and has strong linkages with other WHO guidance (ICF; the WHO application of International Classification of Diseases‐10 to deaths during pregnancy, childbirth and the puerperium: ICD Maternal Mortality [ICD‐MM]; continuum of care; and quality of care).^31^, ^32^, ^33^

## 6 Key Concepts in The Conceptual Framework (See Figure 1)

As explained in the introduction, maternal morbidity first refers to “any health condition attributed to and/or complicating childbirth.” The categories used for the **health conditions** (bottom left, pink box) are from ICD‐MM and include obstetric morbidities, medical morbidities, and injuries.^33^ The first three categories include a total of 121 maternal morbidity conditions as reported within the maternal morbidity matrix mentioned earlier.^6^ It is worth noting that complications associated with surgical care and medical management, including cesarean delivery and episiotomy complications, are incorporated in obstetric conditions. Using these comprehensive categories and ICD‐10 coding to report on conditions will facilitate the consistent reporting and analysis of maternal morbidity diagnoses. To these we have added fetal/infant morbidities in view of their many negative linkages with maternal morbidity outcomes and maternal well‐being.

The immediate **outcomes of maternal conditions** form a continuum starting with full recovery, maternal morbidity (whether short term or long term), potentially life‐threatening conditions, maternal near miss (women who nearly died), or maternal deaths. While individual women can progress from maternal morbidity to full recovery, near miss, or death, retrospective reporting of the final outcomes is best done using mutually exclusive categories. Except for maternal death, each of these conditions can have a negative or, in some cases, positive **impact on functioning and well‐being** of individual women. For example, life‐threatening postpartum hemorrhage and its associated impact on hemoglobin levels can lead to a loss of women's productivity; whereas the loss of a baby will affect the psychological well‐being of a mother, but can also bring a couple or a family closer together in facing adversity. Pregnancy, childbirth, and the lived experience of the postnatal period may also have an independent impact on health, social, and economic functioning and well‐being, whether a woman has recovered or not, but this framework assumes that the more severe the morbidity the higher the risk or probability of an adverse effect on functioning and well‐being.^34^

The **reproductive health cycle** (represented by an ellipse) is placed at the center of the framework, linking the different stages of pregnancy, labor, and childbirth to the puerperium, and pre‐reproductive life to the postreproductive part of the life cycle. A maternal morbidity can start at any time during pregnancy, childbirth, or after pregnancy, and it may be self‐limiting, or may continue. This period is further linked to the women's health status before becoming fertile (nutrition, age at menarche, pre‐existing illnesses or disabilities) and influences the postreproductive and postmenopausal periods. In addition, the interval between pregnancies is an important risk factor for maternal morbidity for women who have more than one pregnancy. Intervals that are too short (5 months or less) or too long (longer than 59 months) have both been associated with complications.^35^ Ethnographic studies have shown the importance of fertility and reproduction to women and their partners, particularly in high‐fertility settings; for this reason, many women become pregnant again even though they might not have recovered from a previous pregnancy.^36^

**External factors** are at the top of the framework, represented by a circle with four rings, and include laws and policies, health systems and quality of care, the pre‐existing socioeconomic status of women, and the health status of women. These interact with the reproductive health cycle, and influence women's risks of becoming pregnant, getting unwell during pregnancy, and complications becoming serious or being eliminated.

The **health system** influences the likelihood and severity of maternal morbidity in the same ways that it influences the reduction of maternal mortality, by preventing complications and by reducing the three delays in: (1) deciding to seek care; (2) reaching the appropriate level of care; and (3) receiving the appropriate treatment. Not all conditions, signs, and symptoms that complicate pregnancies are potentially lethal, however, and many can be treated at the primary healthcare level, especially when they are chronic (see Firoz et al.^7^ in this Supplement). In addition, early detection of noncommunicable diseases and interventions targeting lifestyle factors before conception, during pregnancy, and after pregnancy can prevent other adverse events for women and infants. Diabetes and chronic hypertension, for instance, can both lead to adverse outcomes during pregnancy and delivery, and are risk factors for increased cardiovascular diseases later in life for women.^37^ This is why a life‐cycle approach to women's health care is necessary. The recent WHO quality‐of‐care model highlights the importance, to improve health outcomes, of evidence‐based practices, information systems, referral systems, competent staff, and appropriate facilities, as well as of the perceptions of women about their care.^31^

Many observational studies have demonstrated that women from disadvantaged or poor **socioeconomic backgrounds** do not access reproductive health services at the same level as richer women^38^; and while there is very limited evidence of differences in morbidity incidence risks between disadvantaged and advantaged women, research indicates that disadvantaged women may have more serious adverse pregnancy and health outcomes, such as near‐miss events.^39^ These socioeconomic determinants include education,^40^ occupation,^41^ ethnicity,^41^ and wealth,^39^ as well as issues related to structural violence toward women, and the status of women in their society.

**Laws** that regulate women's work such as maternity leave, and laws concerned with reproductive rights, including access to family planning and abortion, also influence both the risk of morbidity and the chance of recovery postpartum. Similarly, **policies** that invest in improving the social determinants of health (such as female education) and facilitate women's access to health care help reduce both the occurrence of morbidity, and improve access to treatment.^2^ In some settings, women with unintended pregnancies have been found to delay accessing antenatal care and to make fewer visits. There is also evidence, albeit mostly from high‐income settings, that women with unintended pregnancies are also more likely to have depression or anxiety after childbirth.^42^ Finally, the ability offered by social protection to rest and recover after childbirth, particularly when there has been a complication, is paramount to avoiding further adversity.^43^

Health status refers to the underlying or contributing health conditions that the woman may have at the time of conception and pregnancy. The MMWG data collection tool documents, for example, self‐reported violence exposure, obesity, being sexually satisfied, HIV, and substance abuse as well as reproductive risk factors such as age and parity. Using the tool, a high prevalence of obesity (antenatal: 34.9%, postpartum: 22.6%), sexual dissatisfaction (antenatal: 34.4%, postpartum: 21.5%), and exposure to violence (antenatal: 12.8%, postpartum: 11.0%) was found in women presenting for antenatal and postpartum care in Jamaica, Kenya, and Malawi.^44^ Sexual dissatisfaction and living in urban settings were the only two remaining risk factors associated with maternal morbidity in a multivariate analysis using data from the three countries. Globally, the increase in obesity among women of reproductive age is of concern, given its association with gestational diabetes, pre‐eclampsia, and stillbirths. Age—being particularly young or particularly old—is also important, as well as parity.^41^ A list of past obstetric, medical, and social history conditions is included in Chou et al.^6^

Box 1 illustrates the applicability of the framework using a real‐life case study.

Box 1 **Testing the framework using a real‐life case study.**

1 Researchers’ qualitative accounts of women with eclampsia, and for whom there is longitudinal information on more than one pregnancy, can be used to illustrate the applicability of the framework and the different concepts included.^43^ This uneducated woman lived in a rural area of Burkina Faso (**socioeconomic status**). Because she had no formal employment, she had very limited access to social and financial risk protection (**laws and policies**). She farmed her husband's land with her co‐wife and husband. While there is no information on her pre‐reproductive life cycle, we know that she had one previous pregnancy, that a child had died and that she had remarried (**reproductive life cycle**). During her second pregnancy, she became ill with malaria (**medical morbidity, non‐severe)** and eclampsia (**obstetric morbidity, maternal near miss)**. Her second baby died at birth (**fetal/infant mortalities**). After pregnancy, she continued to be unwell, having developed chronic hypertension (**medical condition, puerperium**). She also had depression linked to the loss of her second baby and, being unwell, was unable to perform all of her routine household tasks (**impact on functioning and well‐being**) despite having a supportive family. Because she was poor, she was able to receive only intermittent treatment during the following months, and her **health status** did not improve sufficiently. Because her local health center did not have the type of contraception that she wanted or needed, and against the advice given to her to delay pregnancy, she fell pregnant again (**health system and quality of care**). Sadly, she died during her third pregnancy (**maternal death**). This case was one of many cases of morbidity in a larger cohort study.^45^ How to measure different facets of maternal morbidity comprehensively remains a challenge.

Measurement efforts and interventions to reduce maternal mortality have primarily focused on the period around childbirth, skilled birth attendance, and emergency obstetric care. While these are still very relevant today, we hope that this framework will help researchers, providers, and policy makers recognize where the gaps in knowledge on maternal morbidity exist so that comprehensive research is conducted and better services or policies are provided to reduce its burden. From a research perspective, there is an urgent need for detailed and comprehensive longitudinal studies of women from early pregnancy through the extended postpartum period, to understand how health and symptoms and signs of ill health change during this reproductive period. With respect to interventions, the framework suggests that there may be gaps in healthcare provision for women who have chronic conditions and are about to conceive. Women also require a continuity of care at the primary healthcare level beyond the customary 6 weeks postpartum. Finally, adequate social protection policies and laws are needed so that women can use preventive and curative services when they need them, and recover from illnesses and/or disabilities.

## Author Contributions

DC and MB prepared the first draft of the figure for the conceptual framework, as discussed and amended by the MMWG members. VF wrote the first draft of the paper and made subsequent revisions. LS, MB, and DC commented on drafts. LS conceptualized the maternal morbidity measurement initiative. All authors read and approved the final manuscript.

## MMWG Members

Kelli Barbour, Jose G. Cecatti, Maria L. Costa, Sara Cottler, Olubukola Fawole, Tabassum Firoz, Luis Gadama, Atf Ghérissi, Gathari N. Gichuhi, Gill Gyte, Michelle Hindin, Anoma Jayathilaka, Amanda Kalamar, Marge Koblinsky, Yacouba Kone, Nenad Kostanjsek, Isabelle Lange, Laura A. Magee, Arvind Mathur, Affette McCaw‐Binns, Mark Morgan, Stephen Munjanja, Max Petzold, Elizabeth Sullivan, Frank Taulo, Özge Tunçalp, Rachel Vanderkruik, Peter von Dadelszen.

## Conflicts of Interest

The authors declare no conflicts of interest.
